# Cognitive Impairment in Patients with Glioma: Mechanisms, Assessment, and Emerging Therapeutic Strategies

**DOI:** 10.3390/cancers18121865

**Published:** 2026-06-07

**Authors:** Katarzyna Piec, Maciej Blok, Magdalena Adamczak-Sobczak, Izabela Zarębska, Maciej Harat

**Affiliations:** 1 Department of Neurooncology and Radiosurgery, Franciszek Lukaszczyk Memorial Oncology Center, 85-796 Bydgoszcz, Poland; maciej.blok@pbs.edu.pl (M.B.); adamczak-sobczakm@co.bydgoszcz.pl (M.A.-S.); zarebskai@co.bydgoszcz.pl (I.Z.); maciej.harat@pbs.edu.pl (M.H.); 2 Faculty of Medicine, Bydgoszcz University of Science and Technology, 85-796 Bydgoszcz, Poland

**Keywords:** glioblastoma, glioma, cognitive decline, surgery, chemotherapy, radiotherapy, quality of life

## Abstract

**Aim:** This review summarizes the current evidence on cognitive impairment in patients with glioma, with particular emphasis on the underlying mechanisms, methods for objective assessment of cognitive deficits, and currently available therapeutic strategies. **Methods:** Relevant literature was identified through searches of the PubMed, Cochrane Library, and Google Scholar databases. Studies addressing the mechanisms, assessment, and management of cognitive impairment in patients with glioma published between January 2000 and February 2026 were reviewed to summarize current evidence and emerging therapeutic strategies. A structured literature search and study selection process was performed. **Results:** Cognitive deficits represent a heterogeneous clinical problem affecting the majority of patients diagnosed with gliomas. These impairments are multifactorial in origin, resulting both from direct infiltration of white matter by the tumor process and from the cumulative toxicity of treatment modalities, including radiotherapy, chemotherapy, corticosteroid therapy, and long-term use of antiseizure medications. Analysis of the current literature confirms that modern radiotherapy techniques (e.g., hippocampal-sparing approaches) and isocitrate dehydrogenase (IDH)-targeted therapies, such as vorasidenib, may significantly delay cognitive decline. Standardized neuropsychological batteries for the objective assessment of these disturbances remain the preferred tools recommended by the International Cognition and Cancer Task Force (ICCTF). Regarding therapeutic interventions, potential benefits have been demonstrated for supportive pharmacotherapy (including memantine), while individualized neuropsychological rehabilitation has also been shown to improve patients’ quality of life.

## Simple Summary

Patients with glioma frequently experience cognitive impairment, which can affect memory, attention, and executive functions, significantly reducing quality of life. These deficits arise not only from tumor growth but also from treatment-related factors such as surgery, radiotherapy, and chemotherapy. This review summarizes current knowledge on the mechanisms underlying cognitive dysfunction in glioma, methods for its assessment, and available therapeutic strategies. Particular emphasis is placed on early detection, standardized neuropsychological testing, and the integration of cognitive outcomes into routine clinical care. Improving cognitive function is essential for maintaining independence and optimizing long-term outcomes in patients with glioma.

## 1. Introduction

Gliomas are the most common group of primary tumors of the central nervous system in adults and are characterized by infiltrative growth and marked biological and clinical heterogeneity. Glioblastoma (GBM) is one of the most aggressive malignant tumors, with a median overall survival of approximately 15 months [1]. Advances in neurosurgical techniques, precision radiotherapy, and personalized oncological treatment have led to a significant prolongation of patient survival over recent decades, particularly among individuals with low-grade gliomas and tumors harboring favorable molecular markers. As survival outcomes have improved, preserving health-related quality of life (HRQoL) has become a major challenge in modern neuro-oncology, with cognitive functioning representing one of its key determinants [2,3].

Cognitive impairment occurs in up to 90% of patients with gliomas at various stages of the therapeutic process [4]. These disturbances are not merely a direct consequence of tumor mass effect, but rather result from a complex interaction between tumor biology, mechanisms of brain plasticity, and iatrogenic effects of aggressive multimodal treatment [2,3,5]. Deficits involving memory, attention, executive functions, and processing speed substantially affect patients’ ability to return to work, maintain social roles, and even adhere to therapeutic recommendations.

Despite their clinical relevance, assessment and monitoring of cognitive function in routine neuro-oncological practice remain challenging due to the lack of standardized approaches and the confounding effects of treatment-related complications and polypharmacy [6,7].

**Figure A1:**
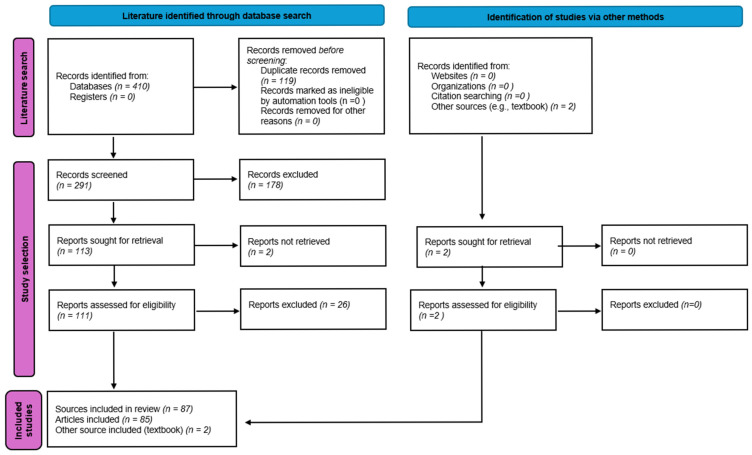
Flow diagram summarizing the literature identification, screening, eligibility assessment, and study selection process used in this review. Publications were retrieved from PubMed, Cochrane Library, and Google Scholar databases and evaluated for relevance to the mechanisms, assessment, and management of cognitive impairment in patients with glioma. **Abbreviations:** n, number of records/studies. **Search Strategy** A comprehensive literature search was conducted in the PubMed, Cochrane Library, and Google Scholar databases. Publications in English and Polish published between January 2000 and February 2026 were considered, with particular emphasis on phase II and III clinical trials, meta-analyses, and recommendations issued by international expert groups. The following keywords and their combinations, using Boolean operators (AND, OR), were applied: glioma, glioblastoma, brain tumor; cognitive impairment, neurocognitive dysfunction, executive functions; radiotherapy-induced cognitive decline, chemobrain, temozolomide; cognitive rehabilitation, Goal Management Training, memantine, and donepezil. The analysis included studies involving adult patients with gliomas (WHO grades 2–4), addressing the effects of oncological treatment (surgery, radiotherapy, and chemotherapy) on neuropsychological status, as well as diagnostic and therapeutic interventions, including pharmacological and non-pharmacological approaches. Case reports, animal studies (unless addressing key biological mechanisms), and non-peer-reviewed publications were excluded. Study selection was performed using a structured, stepwise screening process guided by PRISMA methodology. The initial search identified 410 records. After removal of duplicates (*n* = 119) and screening of titles and abstracts, 111 full-text articles were assessed for eligibility. Ultimately, 87 sources were included in the final review, comprising 85 articles and two textbook.

This review summarizes current knowledge on the mechanisms underlying cognitive impairment in patients with glioma, discusses the impact of major treatment modalities, and outlines contemporary approaches to cognitive assessment and rehabilitation. The literature search and study selection process are presented in Figure A1.

**Figure 1:**
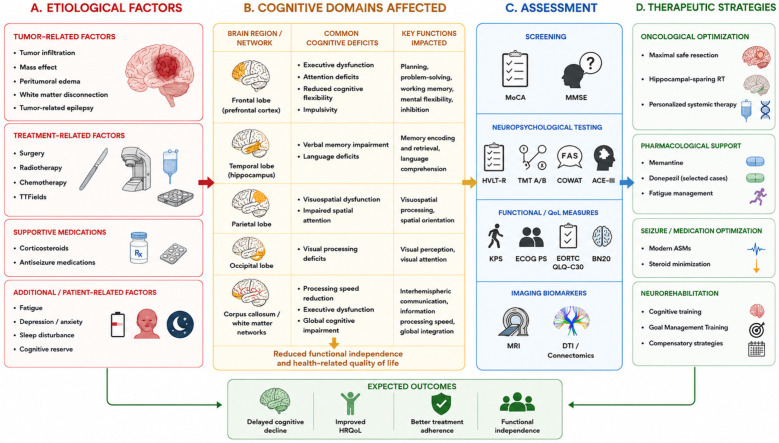
Overview of cognitive impairment in patients with glioma, including etiological factors, affected cognitive domains, assessment strategies, and therapeutic interventions. Tumor-related factors, treatment-related toxicity, supportive medications, and patient-related factors contribute to dysfunction across multiple cognitive domains. The figure summarizes the relationship between affected brain regions/networks and common cognitive deficits, current approaches to cognitive assessment, and available therapeutic strategies aimed at preserving cognitive functioning and quality of life. Created with BioRender.com. **Abbreviations:** ACE-III, Addenbrooke’s Cognitive Examination III; ASM, antiseizure medication; BN20, EORTC Quality of Life Questionnaire Brain Cancer Module; COWAT, Controlled Oral Word Association Test; DTI, diffusion tensor imaging; ECOG PS, Eastern Cooperative Oncology Group Performance Status; EORTC QLQ-C30, European Organisation for Research and Treatment of Cancer Quality of Life Questionnaire Core 30; HVLT-R, Hopkins Verbal Learning Test-Revised; HRQoL, health-related quality of life; KPS, Karnofsky Performance Status; MMSE, Mini-Mental State Examination; MoCA, Montreal Cognitive Assessment; MRI, magnetic resonance imaging; RT, radiotherapy; TMT, Trail Making Test; TTFields, Tumor Treating Fields.

The multifactorial mechanisms, clinical assessment, and management pathways of cognitive impairment in patients with glioma are summarized in Figure 1.

## 2. Etiology of Cognitive Impairment

Cognitive impairment in patients with glioma is multifactorial and reflects the combined effects of tumor-related mechanisms, treatment-associated toxicity, epilepsy and antiseizure medications, glucocorticosteroid therapy, and psychological factors.

### 2.1. Tumor-Related Factors

The primary cause of neuropsychological deficits in patients with gliomas is the direct presence of the tumor mass and its destructive effect on brain architecture. In contrast to vascular lesions, gliomas affect cognitive functioning through several coexisting mechanisms:

#### 2.1.1. Location and Mass Effect

Direct compression of eloquent cortical areas (e.g., speech or planning centers) is associated with their dysfunction. In addition, progressive mass effect causes displacement of brain structures and increased intracranial pressure, which clinically may manifest as generalized slowing of thought processes, impaired attention, and increasing apathy.

#### 2.1.2. Infiltration of White Matter Tracts

Gliomas frequently infiltrate white matter pathways, disrupting communication between distant cortical regions and contributing to network-level cognitive dysfunction (disconnection syndrome) [8,9].

#### 2.1.3. Peritumoral Edema and Blood–Brain Barrier Disruption

Release of proangiogenic factors (e.g., vascular endothelial growth factor, VEGF) may contribute to vascular leakage and the development of vasogenic edema. Accumulation of fluid in the extracellular space disrupts synaptic transmission and the metabolism of healthy neurons, representing one of the causes of sudden cognitive deterioration during tumor progression.

#### 2.1.4. Glutamatergic Excitotoxicity

Glioma cells release glutamate into the extracellular space, leading to excitotoxic neuronal injury through excessive NMDA receptor activation. This mechanism may contribute to cognitive decline, particularly in memory-related domains [10].

#### 2.1.5. Tumor-Related Epilepsy

Gliomas induce changes in the chemical microenvironment of the cortex, leading to neuronal hyperexcitability. Both epileptic seizures and subclinical interictal discharges act as “noise” within the cognitive system, reducing the efficiency of attention and working memory [11].

### 2.2. Treatment-Related Factors

#### 2.2.1. Surgical Treatment

Traditionally, the assessment of surgical outcomes in patients with GBM has focused on technical endpoints, such as volumetric analysis of residual tumor burden, and clinical measures including progression-free survival and overall survival. Other outcomes, including neurological deficits and deterioration in cognitive, language, or functional abilities, may be more difficult to assess objectively, quantitatively, and comprehensively.

According to the literature, up to 75% of patients with GBM may present cognitive impairment prior to surgery [12,13]. In many patients, surgical resection may contribute to immediate improvement in cognitive functioning. This effect may result from reduction in mass effect through decreased compression of healthy cortical and subcortical structures, reduction in vasogenic edema, improved perfusion of peritumoral regions, and better seizure control [14].

Modern surgical management of gliomas increasingly includes awake craniotomy with intraoperative functional mapping, particularly in patients with tumors located near eloquent brain regions. This approach enables real-time assessment not only of language, but also of executive functions, calculation, and emotion recognition, thereby reducing the risk of permanent cognitive sequelae [15,16]. Intraoperative brain mapping facilitates identification of functionally essential cortical and subcortical regions that should be preserved during resection [17], while neuronavigation improves surgical accuracy. Although these techniques often allow a greater extent of safe resection and better preservation of neurological function, a certain risk of postoperative cognitive decline remains [18].

Standardization of key perioperative outcome measures is of major importance in this patient population. The Personalized Interventions and Outcomes in Neurosurgical Oncology Research (PIONEER) consortium and the Response Assessment in Neuro-Oncology (RANO) working group have undertaken efforts to improve reporting standards, clinical research methodology, and outcome assessment in glioma surgery [19].

#### 2.2.2. Radiotherapy

Radiotherapy, used either as first-line treatment or as adjuvant therapy following surgical resection, remains a cornerstone of glioma management. The therapeutic effectiveness of radiotherapy is based primarily on the induction of DNA damage, particularly double-strand breaks, which impair the ability of tumor cells to proliferate and survive. Compared with normal brain tissue, glioma cells often exhibit defective DNA repair mechanisms and increased genomic instability, making them more susceptible to radiation-induced injury. Fractionated treatment further allows partial recovery of surrounding healthy tissues while maintaining cumulative damage within the tumor [1,20,21]. Radiation-based treatment strategies in glioma include external beam radiotherapy, currently most commonly delivered using intensity-modulated radiation therapy (IMRT), less frequently proton therapy in selected primary tumors, and stereotactic radiosurgery/stereotactic radiotherapy in the recurrent setting [5,22].

For central nervous system tumors, IMRT is typically administered over 5–6 weeks, with a total dose of 54–60 Gy delivered in daily fractions of 1.8–2 Gy, five days per week. The effects of ionizing radiation are not limited to tumor tissue but also involve surrounding healthy brain parenchyma. Radiation injury is primarily mediated through cell death, particularly in proliferating cell populations, as well as through tissue repair and remodeling processes occurring within irradiated normal tissues. Adverse effects depend on the total dose, fractionation schedule, anatomical location, and irradiated volume. Previous chemotherapy and prior irradiation further increase the risk of radiation-induced injury. Neurotoxicity affecting healthy brain tissue within treatment margins is considered one of the potential causes of long-term cognitive decline.

The pathophysiology of radiation-induced brain injury is complex and evolves over time, allowing classification into three phases: acute toxicity, early delayed toxicity (up to 6 months after RT), and late toxicity [23].

During the acute and early delayed phases, the most common manifestations include somnolence and attention deficits related to transient vasogenic edema and impaired myelin synthesis. These symptoms are usually reversible and often respond well to corticosteroid therapy. The late phase, characterized by irreversible damage, generally begins approximately 6 months after RT and may include radiation-induced contrast enhancement (RICE), associated with perivascular inflammation and focal edema secondary to radiation vasculopathy in high-dose regions. The frontal and temporal lobes, particularly near the frontal horns of the lateral ventricles, are among the regions most susceptible to RICE.

In addition, radiation may induce leukoencephalopathy, especially in patients older than 65 years and in those with pre-existing leukoaraiosis. This complication usually develops 1–2 years after RT, particularly when large brain volumes have been irradiated. Clinically, it may present with progressive cognitive impairment or seizures of variable semiology. White matter injury may lead to psychomotor slowing and executive dysfunction [24]. Early RT-related neurotoxicity is thought to result mainly from microvascular injury and suppression of neurogenesis. In contrast, late cognitive deficits are primarily associated with progressive demyelination and ischemic necrosis of white matter, leading to disruption of functional connectivity within the brain connectome [24].

Clinical studies have shown that limiting radiation dose to hippocampal stem cell niches significantly preserves memory performance as assessed by the Hopkins Verbal Learning Test–Revised (HVLT-R) [25,26]. These findings contributed to the development of hippocampal avoidance whole-brain radiotherapy (HA-WBRT). It should also be emphasized that advanced age and concomitant vascular comorbidities (e.g., hypertension, diabetes mellitus) substantially reduce cerebrovascular reserve, thereby accelerating radiation-induced neurodegenerative processes [24].

Traditional concerns regarding delayed RT neurotoxicity in patients with low-grade glioma (LGG) are currently being re-evaluated in light of modern clinical evidence. Long-term analysis of the phase III NCCTG 86-72-51 trial demonstrated that, among patients with abnormal baseline screening cognitive function measured using the Mini–Mental State Examination (MMSE) or with neurological deficits, clinical improvement after radiotherapy was observed more frequently than further deterioration [27]. This suggests that tumor control and reduction in mass effect may provide cognitive benefits that outweigh treatment-related injury to normal brain tissue.

Contemporary techniques such as IMRT, hippocampal avoidance radiotherapy (HA-RT), and emerging adaptive radiotherapy may further improve sparing of regions involved in neurogenesis and episodic memory. Available data suggest that, in the modern management of LGG, cognitive deterioration may be driven less by radiotherapy itself and more by disease-related factors, including infiltrative tumor growth, drug-resistant epilepsy, and chronic use of antiepileptic drugs or corticosteroids. These conclusions support the hypothesis proposed by Koutsarnakis et al., indicating the need to redefine neurotoxicity risk in the era of precision medicine [28].

Increasing attention has also been paid to the role of radiotherapy-induced inflammatory changes within the central nervous system [29]. Emerging evidence suggests that chronic microglial activation and inflammatory mediator release may contribute to RT-related cognitive dysfunction. In addition to its role in DNA damage repair, activation of poly(ADP-ribose) polymerase (PARP) may further enhance these inflammatory pathways. Consequently, PARP inhibitors are currently being investigated in combination with radiotherapy in patients with GBM [23]. Other experimental approaches, including phytochemicals, nanocarrier-based drug delivery systems, and microRNA-targeted therapies are also being explored to reduce radiation-related neurotoxicity while improving blood–brain barrier penetration [30].

#### 2.2.3. Chemotherapy

The impact of chemotherapy on cognitive functioning in patients with cancer, commonly referred to as chemotherapy-induced cognitive impairment (CICI) or “chemobrain”, represents an important diagnostic challenge because cytotoxic effects often overlap with the direct consequences of the tumor itself and prior radiotherapy. Several mechanisms contributing to chemotherapy-induced cognitive impairment overlap with those observed after radiotherapy [31,32].

Combined chemoradiotherapy is frequently used as a standard treatment approach in most patients with gliomas. Antineoplastic agents may impair cognitive functioning through several mechanisms, including hippocampal injury, impaired neurogenesis, white matter damage, hypothalamic–pituitary–adrenal axis dysregulation, and vascular or inflammatory alterations [21,33,34].

##### Temozolomide

The backbone of chemotherapy for GBM is temozolomide (TMZ), which is generally well tolerated, with adverse events being predominantly hematological and usually manageable [35].

As a small-molecule alkylating agent, TMZ readily crosses the blood–brain barrier, which underlies its antitumor efficacy but may also expose healthy brain tissue to potential toxicity. TMZ methylates DNA, leading to the death of rapidly proliferating tumor cells [36]. However it may also affect non-dividing or slowly dividing cells, including glial progenitors and other vulnerable neural cell populations, thereby contributing to neurotoxicity and cognitive dysfunction [37]. TMZ significantly improves overall survival and progression-free survival, but treatment may also be associated with fatigue, impaired concentration, and other cognitive complaints [5]. In addition, higher cumulative exposure has been associated with an increased risk of neurotoxicity [38].

It is also noteworthy that patients with MGMT promoter methylation, who typically derive greater therapeutic benefit from TMZ, may paradoxically present more pronounced cognitive deficits over time. This observation may partly reflect longer survival and greater exposure to delayed treatment-related effects [39]. Clinical studies have suggested that, compared with radiotherapy alone, combined chemoradiotherapy may be associated with more severe cognitive impairment [40]. Preclinical data further support this observation. Gan et al. reported that radiotherapy combined with concurrent TMZ induced damage to normal brain tissue in mice, resulting in anxiety-like behavior and cognitive impairment [41]. Similarly, Dey et al. demonstrated that combined RT and TMZ exacerbated treatment-related brain injury and induced depressive-like behaviors in mice, potentially through disruption of serotonergic signaling pathways [42]. Taken together, these findings suggest that combined chemoradiotherapy may exacerbate brain injury and cognitive dysfunction, particularly in long-term survivors.

##### Procarbazine–Lomustine–Vincristine (PCV) Regimen

Other chemotherapeutic agents, such as nitrosoureas, may potentially exhibit a less favorable neurotoxicity profile than TMZ [43]. The impact of the PCV regimen (procarbazine, lomustine, vincristine) on cognitive functioning has been evaluated in several pivotal phase III clinical trials involving patients with LGG and anaplastic oligodendroglioma.

In the RTOG 9802 trial, the addition of PCV chemotherapy to radiotherapy in patients with WHO grade 2 glioma improved progression-free survival. Importantly, this combined treatment strategy was not associated with a statistically significant decline in MMSE scores compared with radiotherapy alone during 5 years of follow-up [44]. Similarly, results from the RTOG 9402 trial, which evaluated patients with anaplastic oligodendroglioma (WHO grade 3) treated with RT alone or RT combined with PCV, showed that among surviving patients, MMSE scores initially remained stable in both treatment arms, suggesting relatively preserved cognitive functioning after completion of therapy. In contrast, quality of life assessed using the B-QOL questionnaire gradually declined in both groups [45]. Cognitive deterioration was observed mainly in patients who developed tumor progression or during the final year of life, suggesting that disease progression rather than treatment itself may be the principal determinant of cognitive decline.

#### 2.2.4. Bevacizumab

The impact of bevacizumab (an anti-VEGF monoclonal antibody) on cognitive functioning remains controversial.

On the one hand, owing to its potent anti-edema effects and its ability to reduce glucocorticosteroid requirements (steroid-sparing effect), bevacizumab often leads to rapid symptomatic improvement in neurological status and overall HRQoL, as reported, among others, in the phase III AVAglio trial [46].

On the other hand, objective neuropsychological assessments performed within the parallel RTOG 0825 trial in patients with high-grade glioma (HGG) demonstrated that individuals receiving bevacizumab more frequently experienced accelerated decline in executive functioning and reduced processing speed compared with the placebo group. The median time to objective deterioration in neurocognitive function was only 1.22 months in the bevacizumab arm [47]. This rapid decline primarily involved working memory, executive functions, and processing speed.

These findings suggest that prolonged inhibition of vascular endothelial growth factor (VEGF) may adversely affect neurogenesis and the homeostasis of healthy brain tissue. However, it should be emphasized that in the setting of recurrent glioma or treatment of symptomatic radiation necrosis, the clinical benefits associated with control of vasogenic edema often outweigh the potential risk of neurotoxicity. In such cases, reduction in intracranial pressure may contribute to stabilization or transient improvement of cognitive status [47,48].

#### 2.2.5. Vorasidenib

The introduction of vorasidenib, a selective inhibitor of mutant IDH1/2 enzymes, represents a novel therapeutic strategy in the management of LGG, aimed at modifying the natural course of disease while attempting to preserve neuropsychological functioning. Results of the phase III INDIGO trial demonstrated that treatment with vorasidenib significantly prolonged progression-free survival and, importantly from a clinical perspective, delayed the need to initiate more toxic treatment modalities such as radiotherapy or alkylating chemotherapy [49]. Although preliminary analyses of HRQoL did not demonstrate significant deterioration in cognitive functioning in the treatment group compared with placebo, these findings should be interpreted with caution in the long term. The current follow-up period in the INDIGO trial remains relatively short in relation to the expected survival of patients with IDH-mutant gliomas.

Although currently available data are reassuring, longer follow-up is needed to evaluate the long-term neurocognitive safety of IDH inhibitors. [49].

#### 2.2.6. Immunotherapy

Immunotherapeutic strategies, including immune checkpoint inhibitors, therapeutic vaccines, oncolytic viruses, and adoptive cellular therapies, represent an active area of investigation in glioma treatment. Despite encouraging preclinical findings, clinical results have thus far been limited, partly due to the highly immunosuppressive tumor microenvironment and the unique immune characteristics of the central nervous system. At present, evidence regarding the impact of immunotherapy on cognitive functioning in patients with glioma remains scarce. Nevertheless, improved tumor control and reduced reliance on corticosteroids may potentially contribute to preservation of neurocognitive function. Further studies are needed to clarify the role of immunotherapy in long-term cognitive outcomes among glioma survivors [50].

#### 2.2.7. Tumor Treating Fields (TTFields)

In recent years, the standard treatment of GBM has been expanded to include Tumor Treating Fields (TTFields), a non-invasive modality using low-intensity, intermediate-frequency alternating electric fields (100–300 kHz). The mechanism of action of TTFields is based on selective disruption of mitosis in rapidly dividing tumor cells through interference with microtubule polarization and mitotic spindle formation, while largely preserving the integrity of non-dividing neurons.

One of the attractive features of TTFields therapy is its minimal invasiveness and lack of systemic adverse effects. This is particularly relevant in the recurrent disease setting, where patients are often exposed to multiple prior treatments, including chemotherapy, repeat surgery, and/or re-irradiation.

The EF-11 and EF-14 trials demonstrated that TTFields therapy is generally safe with respect to cognitive functioning in patients with GBM. The EF-11 study, which compared TTFields with physician’s choice chemotherapy in patients with recurrent disease, showed improved patient-reported well-being associated with fewer adverse effects due to avoidance of chemotherapy-related toxicity, although without a significant improvement in overall survival [51]. The EF-14 trial, which evaluated the addition of TTFields to standard TMZ maintenance therapy in newly diagnosed GBM, similarly demonstrated no detrimental effect on cognitive functioning compared with standard treatment alone. In addition, patients receiving TTFields reported comparable or slightly better quality of life in domains such as emotional and physical functioning than those treated with TMZ alone [52,53]. Despite evidence supporting its clinical efficacy, the availability and implementation of TTFields remain heterogeneous across healthcare systems worldwide.

### 2.3. Additional Factors

#### 2.3.1. Corticosteroid Therapy

Dexamethasone remains the standard treatment for peritumoral edema and for alleviating symptoms related to increased intracranial pressure. Although its short-term use often leads to apparent improvement in cognitive functioning through reduction in mass effect and decompression of healthy brain structures, prolonged corticosteroid exposure is associated with a substantial risk of iatrogenic neuropsychological deficits.

The principal target of adverse glucocorticoid effects within the central nervous system is the limbic system, particularly the hippocampus. Prolonged exposure to high doses of dexamethasone has been associated with impaired neurogenesis, structural changes within hippocampal networks, and disturbances in memory and learning processes [54,55]. Long-term corticosteroid therapy may also adversely affect attention and executive functioning, contributing to cognitive impairment in patients with glioma [56]. Clinically, this may manifest as specific deficits in declarative memory that can be misinterpreted as tumor progression.

Corticosteroid therapy may also contribute to attentional and executive dysfunction as well as mood disturbances, including anxiety, irritability, insomnia, and depressive symptoms. These effects may further complicate the interpretation of neuropsychological assessments [13].

It should be emphasized that when interpreting cognitive test results (e.g., before surgery or during radiotherapy), the current dose of dexamethasone should always be documented, as it may substantially influence the patient’s neurocognitive profile.

#### 2.3.2. Epilepsy and Antiseizure Medications

Tumor-related epilepsy (TRE) is a common and clinically significant consequence of central nervous system tumors. Seizures occur in approximately 60–90% of patients with gliomas, often necessitating long-term treatment with antiseizure medications (ASMs). TRE is more common in lower-grade gliomas and in tumors harboring IDH mutations, highlighting the important contribution of tumor biology to epileptogenesis [57]. Optimal therapeutic selection is of major importance, as older-generation ASMs may themselves cause clinically relevant neurotoxicity, potentially masking the patient’s true cognitive status.

The addition of ASMs may exacerbate pre-existing symptoms or induce new dysfunctions [3]. Therefore, highly individualized antiseizure management, integrated with oncological treatment strategies, remains a key challenge in neuro-oncological practice.

Older-generation ASMs are associated with a higher risk of cognitive adverse effects and clinically relevant drug–drug interactions, whereas newer-generation agents such as levetiracetam, lacosamide, and lamotrigine generally demonstrate a more favorable cognitive profile and improved tolerability. Nevertheless, behavioral adverse effects may occur and should be monitored during long-term treatment [58,59,60].

A summary table of selected antiseizure medications, including mechanisms of action, potential interactions with TMZ, and effects on cognitive functioning, is presented in Table 1 (adapted from [61]).

## 3. Cognitive Deficit Profile

The spectrum of cognitive impairment in glioma is highly heterogeneous and depends on tumor location, growth dynamics (according to WHO grade), and the extent of the peritumoral edema zone. Unlike sharply circumscribed lesions, infiltrative gliomas disrupt distributed white matter networks, resulting in neuropsychological deficits that often extend beyond the anatomical boundaries of lesions visible on magnetic resonance imaging (MRI).

Available evidence indicates that cognitive dysfunction in patients with GBM is multidomain in nature, involving memory, executive functioning, attention, visuospatial abilities, and language performance [3,62,63]. Tucha et al. [63] demonstrated that a substantial proportion of patients with GBM present impairments in memory, attention, and executive processes even before initiation of oncological treatment. Tumor location remains one of the principal determinants of both the presence and severity of cognitive dysfunction.

Several studies have demonstrated that left-hemispheric gliomas are more frequently associated with deficits in verbal memory, learning, and language functions, whereas right-sided lesions more commonly affect visuospatial abilities and processing speed [7,63]. Tumors involving the frontal lobes primarily predispose patients to deficits in executive functioning, including impaired planning, decision-making, and inhibitory control [64].

Temporal lobe involvement frequently leads to memory impairment and language comprehension difficulties [13]. Comparing the neuropsychological profiles of patients with temporal gliomas, Noll et al. [13] demonstrated that left-sided temporal tumors were associated with slower learning, poorer verbal memory, and deficits in language, executive functioning, and attention, while processing speed remained relatively preserved. In contrast, right-sided temporal gliomas were characterized predominantly by executive dysfunction, impaired learning, reduced verbal memory, slowed processing speed, and diminished fine motor performance.

Tumors located in the parietal and occipital lobes may impair spatial orientation, attentional processes, and cortical visual information processing [65]. Moreover, infiltration of subcortical white matter destabilizes large-scale neuronal networks, leading to widespread deficits in processing speed and inter-network connectivity [2].

Wu et al. demonstrated that gliomas involving the insular region predispose patients to specific cognitive dysfunction, particularly impaired naming performance in confrontation tasks [66].

Another strategically important structure in the generation of cognitive deficits is the corpus callosum (CC), the largest white matter commissure integrating both cerebral hemispheres. Its involvement leads to impaired interhemispheric communication [67]. Hautmann et al., studying patients with GBM infiltrating the corpus callosum, used diffusion tensor imaging to assess fractional anisotropy across three CC segments and found that neurocognitive deficits strongly correlated with reduced microstructural integrity [12]. Visual and lexical memory were associated with the rostrum, executive functioning and working memory with the truncus (body), and processing speed with the splenium of the corpus callosum.

Current evidence suggests that untreated disease leads to rapid progression of location-dependent cognitive deficits due to uncontrolled tumor growth and increasing mass effect. By contrast, implementation of optimal treatment—including maximal safe resection with preservation of eloquent areas, followed by control of tumor proliferation with radiotherapy and chemotherapy—may significantly slow the trajectory of cognitive deterioration. When combined with targeted neurorehabilitation, partial restoration of selected functions may also be achievable [63,68,69].

Given the direct impact of cognitive dysfunction on quality of life, efforts aimed at preserving neuropsychological functioning after treatment must be carefully balanced against the survival benefits associated with more extensive resection and aggressive adjuvant therapy [66,67].

The cognitive consequences of glioma depend not only on tumor location but also on the rate of tumor progression, which influences the brain’s capacity for functional reorganization. This concept is well illustrated by the comparison between low-grade gliomas (LGG) and high-grade gliomas (HGG) [70]. Due to their slow growth, LGGs allow gradual neuroplastic adaptation and reorganization of functional networks, which may explain the relatively preserved cognitive functioning observed in some patients despite involvement of eloquent brain regions [14]. In contrast, HGGs are characterized by rapid growth, progressive edema, and mass effect, limiting compensatory mechanisms and resulting in earlier and more pronounced cognitive impairment. In this setting, disruption of large-scale brain networks may lead to rapid deterioration in executive functioning, attention, and processing speed [9]. Cognitive reserve may further modulate these effects, delaying the clinical manifestation of cognitive deficits despite ongoing structural brain damage [71].

Habets et al. performed neuropsychological assessment in 72 patients with glioma and demonstrated that, compared with healthy controls, preoperative cognitive performance was significantly reduced across all examined domains, particularly in attention (30% of patients) and working memory (20% of patients) [72]. The authors further showed that patients with left-hemispheric GBM were more susceptible to impairment in neurocognitive function. Precise identification of the left frontoparietal network involved in NCF may not only optimize neurosurgical planning but may also be incorporated into cognitive counseling and rehabilitation algorithms [72].

Modern neuro-oncology is increasingly moving away from classical rigid localizationism toward connectome-based models, in which the brain is understood as a complex network of functionally interconnected nodes. Due to their infiltrative growth pattern, gliomas disrupt this network through damage to strategic white matter association pathways. This helps explain why patients often present with cognitive deficits that appear disproportionately extensive relative to the anatomical size of the tumor [73,74].

## 4. Diagnosis and Monitoring

The assessment and longitudinal monitoring of cognitive functioning in patients with glioma represent one of the major challenges of contemporary neuro-oncology. Although there is broad consensus that comprehensive, standardized neuropsychological batteries remain the most reliable tools for objectively detecting subtle deficits in domains such as executive functioning or working memory, routine clinical implementation remains limited by several practical and methodological barriers.

A major obstacle is the lack of unified standards regarding test selection, diagnostic thresholds, and definitions of cognitive impairment. Investigators and clinicians frequently apply heterogeneous criteria for identifying and grading deficits, which substantially limits comparisons across studies and institutions. This issue is further compounded by the absence of an ideal screening instrument combining high sensitivity for early neurocognitive change with brevity and low patient burden during demanding oncological treatment [75]. Another relevant source of heterogeneity is the lack of consistency in follow-up assessment schedules. Differences in testing intervals make it difficult to capture the trajectory of cognitive changes, ranging from transient postoperative effects to delayed radiotherapy-associated decline. Consequently, the development of optimal therapeutic and rehabilitation pathways is often hindered.

To address these limitations, the International Cognition and Cancer Task Force (ICCTF) proposed a minimum core battery for neuro-oncology research [76], based on tests with high sensitivity to deficits commonly observed in brain tumor patients:

- HVLT-R (Hopkins Verbal Learning Test–Revised)—verbal learning and memory;
- Trail Making Test (TMT) Parts A and B—psychomotor speed, attention, and cognitive flexibility;
- COWAT (Controlled Oral Word Association Test)—verbal fluency and executive control [6].

Repeated testing may be affected by practice effects, potentially masking subtle cognitive decline despite the use of alternate test versions. Patients with glioma are frequently burdened by fatigue, neurological symptoms, and treatment-related toxicity. A full neuropsychological evaluation may last 2–3 h, during which exhaustion can significantly influence results, making it difficult to distinguish true cognitive dysfunction from nonspecific somatic burden. For routine clinical purposes, an assessment duration of approximately 30–40 min is often considered optimal, balancing feasibility with adequate clinical sensitivity [77]. Importantly, cognitive deterioration may precede radiological progression on MRI and can constitute an early sign of recurrence. Prompt screening for newly emerging cognitive symptoms may therefore accelerate imaging diagnostics and earlier recognition of disease progression.

Modern neuro-oncology increasingly emphasizes a holistic outcome model, in which progression-free survival is interpreted alongside HRQoL. In patients with glioma, cognitive, neurological, and psychiatric symptoms often overlap, and objective neuropsychological test scores do not always fully reflect real-world functioning. Therefore, formal cognitive assessment should routinely be complemented by measures of global performance status and patient-reported quality of life.

The most widely used tools for global clinical assessment remain performance scales such as:

- Karnofsky Performance Status (KPS);
- ECOG/WHO Performance Status.

These instruments provide rapid quantification of independence and ability to tolerate intensive oncological treatment. A low KPS score (<70) has been shown to be a strong independent predictor of shorter survival and is also associated with more severe cognitive dysfunction [9].

The gold standard instruments for subjective well-being in oncology are questionnaires developed by the European Organisation for Research and Treatment of Cancer (EORTC). In clinical studies, a two-step model is commonly used:

- EORTC QLQ-C30—general oncology module evaluating physical, emotional, role, cognitive, and social functioning, as well as symptoms such as fatigue, pain, nausea, and vomiting;
- EORTC QLQ-BN20—brain tumor-specific module assessing symptoms such as seizures, headaches, drowsiness, communication difficulties, visual deficits, and subjective cognitive decline.

Other relevant quality-of-life instruments include:

- FACT-Br (Functional Assessment of Cancer Therapy–Brain);
- MDASI-BT (MD Anderson Symptom Inventory–Brain Tumor);
- EQ-5D, a generic HRQoL tool frequently used in clinical and health-economic studies.

Commonly used instruments include MMSE, MoCA, ACE-III, CDT, TMT, COWAT, and HVLT-R. Their principal cognitive domains, advantages, and limitations are summarized in Table 2. Among these tools, MoCA generally demonstrates greater sensitivity than MMSE for detecting mild cognitive impairment, whereas HVLT-R, TMT, and COWAT remain core components of many neuro-oncology assessment batteries [78,79].

## 5. Therapeutic Strategies

Optimization and preservation of cognitive functioning in patients with glioma generally rely on a dual-track model that combines non-pharmacological neuropsychological interventions with selected attempts at pharmacological neuroprotection and symptomatic support.

### 5.1. Neuropsychological Rehabilitation

Contemporary cognitive rehabilitation in patients with glioma is generally based on two complementary approaches: restorative and compensatory strategies. Restorative interventions aim to improve impaired cognitive domains through structured training programs that promote neuroplasticity, whereas compensatory strategies focus on adaptation to persistent deficits using external aids and behavioral techniques. Selection of a rehabilitation strategy should be individualized and guided by the patient’s cognitive profile, functional status, treatment-related limitations, and personal goals. Restorative approaches may be most appropriate for patients with mild to moderate deficits and preserved learning capacity, whereas compensatory strategies are often preferred in individuals with persistent impairments. Success should be assessed not only by improvements in neuropsychological test performance but also by functional outcomes, including independence in daily activities, return to work, treatment adherence, and health-related quality of life. Among available approaches, Goal Management Training (GMT) has shown promise in addressing executive dysfunction and improving everyday functioning [80,81].

### 5.2. Supportive Pharmacotherapy

In the search for strategies to mitigate treatment-related neurotoxicity, several agents routinely used in neurodegenerative disorders—particularly Alzheimer’s disease—have been investigated.

#### 5.2.1. Memantine

Memantine, a non-competitive N-methyl-D-aspartate (NMDA) receptor antagonist, is currently the best-studied pharmacological agent in the context of radiation-associated neuroprotection. The pivotal phase III RTOG 0614 trial demonstrated that patients receiving memantine during and after whole-brain radiotherapy (WBRT) for brain metastases experienced significantly less decline in memory function and a longer time to cognitive deterioration [82]. However, the role of memantine in preventing or reducing neurocognitive dysfunction in patients with primary brain tumors, including glioma, remains uncertain. Ongoing clinical trials are evaluating this hypothesis. These include the multicenter randomized phase III MEMENTO trial assessing cognitive outcomes in patients undergoing craniospinal irradiation (CSI) [83], as well as the phase III ACCL 2031 study conducted by the Children’s Oncology Group in pediatric patients receiving cranial radiotherapy.

Importantly, the pilot MEMCRT study (*n* = 30) has already suggested beneficial effects of memantine on processing speed, learning fluency, and fatigue reduction after 12 weeks of treatment [84].

Although the principal rationale for memantine use in glioma remains neuroprotection and delay of radiotherapy-related memory decline, preclinical studies have also suggested that blockade of glutamatergic signaling could theoretically reduce tumor invasiveness by mitigating excitotoxic mechanisms [10]. Given the early stage of evidence and lack of definitive clinical data, memantine should currently be regarded as a supportive rather than disease-modifying intervention.

#### 5.2.2. Donepezil

Donepezil, an acetylcholinesterase inhibitor, has also been investigated as a potential supportive treatment for cognitive dysfunction in patients with brain tumors. Although the evidence base is less consistent than for memantine, some studies have reported improvements in attention, graphomotor speed, and overall quality of life in patients with brain tumors [85], while others failed to demonstrate significant global cognitive benefit [86].

#### 5.2.3. Psychostimulants

Psychostimulants such as methylphenidate and modafinil have been explored as potential treatments for cancer-related fatigue and cognitive dysfunction. Preliminary studies suggest possible improvements in attention, processing speed, executive functioning, and quality of life, particularly in patients with more pronounced baseline deficits [87]. However, current evidence remains limited, and routine use cannot yet be recommended.

## 6. Limitations and Future Directions

The current evidence on cognitive impairment in patients with glioma should be interpreted in light of several important limitations. Available studies are highly heterogeneous with regard to tumor grade, molecular subtype, disease stage, prior treatment exposure, and follow-up duration. Considerable variability also exists in the neuropsychological tools used across studies, ranging from brief screening instruments to comprehensive cognitive batteries, limiting direct comparison of outcomes. Furthermore, differences in the timing of cognitive assessments make it difficult to distinguish transient treatment-related effects from progressive long-term decline. Despite growing interest in cognitive preservation, prospective randomized data evaluating neuroprotective and rehabilitation strategies in patients with primary glioma remain limited.

Nevertheless, several promising developments may improve the assessment and management of cognitive dysfunction in neuro-oncology. Increasing attention is being directed toward connectome-based approaches that integrate diffusion MRI, functional imaging, and network-level brain mapping to better preserve critical cognitive networks during neurosurgical and radiotherapeutic interventions. Further advances in cognition-sparing radiotherapy may allow more individualized protection of vulnerable brain regions beyond the hippocampus.

Pediatric patients constitute a distinct population requiring age-specific cognitive assessment and rehabilitation strategies. Ongoing neurodevelopment, educational functioning, family involvement, and long-term survivorship considerations may substantially influence both the manifestation of cognitive deficits and rehabilitation goals. Because the present review focuses on adult patients with glioma, pediatric populations were beyond its scope and warrant dedicated future studies.

Digital cognitive monitoring represents another emerging area of interest. Remote neuropsychological assessment, smartphone-based testing, and wearable technologies may facilitate earlier detection of subtle cognitive changes and treatment-related toxicity. Integration of these data with imaging findings and molecular biomarkers may further improve individualized patient monitoring.

Artificial intelligence and machine-learning models may also support personalized care by identifying patients at increased risk of cognitive decline based on clinical, radiological, and treatment-related factors. Such approaches could help optimize surveillance strategies and guide early supportive interventions.

From a therapeutic perspective, additional randomized clinical trials are required to clarify the role of neuroprotective agents, including memantine and donepezil, as well as the effectiveness of structured neuropsychological rehabilitation programs. Future studies should incorporate standardized cognitive and quality-of-life endpoints to improve comparability across investigations.

Ultimately, contemporary glioma care is expected to adopt an increasingly multidisciplinary survivorship model involving neuro-oncologists, radiation oncologists, neurosurgeons, neuropsychologists, psychiatrists, rehabilitation specialists, and supportive care teams. In this evolving paradigm, successful treatment will be defined not only by prolonged survival but also by preservation of cognitive functioning, independence, and quality of life.

## 7. Conclusions

Cognitive deficits in patients with glioma are multifactorial in origin. Their pathogenesis is not limited to direct tumor infiltration and mass effect, but is also substantially influenced by iatrogenic consequences of multimodal treatment, including radiotherapy and chemotherapy, as well as supportive pharmacotherapy such as glucocorticosteroids and antiseizure medications.

Contemporary neuro-oncology increasingly recognizes gliomas as disorders affecting large-scale neural networks rather than solely focal lesions, emphasizing the importance of preserving structural and functional brain connectivity [9].

Standardized cognitive assessment remains an important unmet need in routine clinical practice. Early identification and longitudinal monitoring of cognitive deficits may facilitate timely supportive interventions and improve patient-centered care [76].

Current evidence suggests that cognitive preservation should be considered alongside traditional oncological outcomes when planning treatment. Advances in surgical techniques, radiotherapy, and supportive interventions offer opportunities to mitigate treatment-related cognitive decline while maintaining oncological efficacy.

Finally, neuropsychological rehabilitation should be considered an important component of multidisciplinary glioma care. Optimizing cognitive functioning may contribute to improved quality of life, greater independence, and better adherence to complex treatment pathways.

## Figures and Tables

**Table 1:** Cognitive effects, mechanisms of action, and potential oncological interactions of selected antiseizure medications in patients with glioma.

Drug	Principal Mechanism of Action	Cognitive Profile	Potential Interactions with Oncological Therapy (Mainly TMZ)
**Levetiracetam**	SV2A ligand	Favorable cognitive profile; occasional irritability or mood changes.	Minimal interaction potential
**Lacosamide**	Sodium channel modulator	Cognitively neutral; low sedative burden.	Minimal interaction potential.
**Valproic acid**	GABA enhancement; sodium channel blockade	May impair attention; sedation possible.	May inhibit hepatic metabolism and increase hematological toxicity risk during chemotherapy.
**Phenytoin**	Sodium channel blocker	Unfavorable; sedation and cognitive slowing.	Strong enzyme inducer; may reduce serum concentrations of corticosteroids and other drugs.
**Carbamazepine**	Sodium channel blocker	Unfavorable; sedation and cognitive slowing.	Strong CYP3A4 inducer; substantial risk of drug–drug interactions.
**Oxcarbazepine**	Sodium channel blocker	More favorable than carbamazepine; moderate risk of fatigue or dizziness.	Lower interaction risk than carbamazepine, though mild enzyme induction may occur.
**Lamotrigine**	Sodium channel blocker; glutamate modulation	Generally favorable or neutral; may improve mood in selected patients.	Low interaction potential; dose adjustment required with valproic acid.
**Brivaracetam**	High-affinity SV2A ligand.	Favorable; lower risk of behavioral adverse effects than levetiracetam in some studies.	Minimal interaction potential.
**Topiramate**	Mixed mechanism (GABA enhancement, glutamate inhibition)	Higher risk of cognitive adverse effects, particularly word-finding difficulties.	May cause metabolic acidosis; caution required in multimorbid patients.
**Pregabalin**	α2δ calcium channel ligand	Sedation; possible concentration difficulties.	Minimal hepatic interactions.
**Clonazepam/Diazepam**	GABA-A receptor potentiation	Unfavorable during chronic use; sedation and memory impairment.	Risk of dependence and excessive sedation; may confound neurocognitive testing.
**Cannabidiol (CBD)**	Multimodal mechanism	Variable; may improve sleep and anxiety, though somnolence may occur.	Inhibits CYP2C19 and CYP3A4; may increase levels of co-administered drugs.
**Zonisamide**	Sodium and T-type calcium channel blocker	May impair concentration and processing speed in some patients.	Risk of metabolic adverse effects; monitor hydration and renal status.
**Phenobarbital**	GABA-A receptor potentiation	Strongly unfavorable; marked sedation and psychomotor slowing.	Potent enzyme inducer; multiple clinically relevant drug interactions.

**Abbreviations:** TMZ, temozolomide; SV2A, synaptic vesicle glycoprotein 2A; GABA, gamma-aminobutyric acid; CYP, cytochrome P450.

**Table 2:** Commonly used tools for cognitive assessment in patients with glioma: domains evaluated, clinical utility, and limitations.

Test	Type	Main Domains Assessed	Approximate Administration Time	Clinical Strengths	Main Limitations
**MoCA (Montreal Cognitive Assessment)**	Screening	Executive functions, attention, memory, language, visuospatial abilities	10–15 min	High sensitivity for mild cognitive impairment and executive dysfunction; preferred over MMSE in many neuro-oncology settings	Less informative than full neuropsychological batteries; affected by education level
**MMSE (Mini–Mental State Examination)**	Screening	Global cognition, orientation, memory, language	5–10 min	Widely known, rapid, easy to administer, useful for serial bedside assessment	Low sensitivity for frontal/executive deficits; ceiling effect in highly functioning patients
**ACE-III (Addenbrooke’s Cognitive Examination III)**	Extended screening	Attention, memory, verbal fluency, language, visuospatial functioning	15–20 min	Broader cognitive profile than MMSE/MoCA; useful when more detailed screening is needed	Longer administration time; less practical in fatigued patients
**CDT (Clock Drawing Test)**	Brief screening	Visuospatial construction, executive planning, semantic memory	2–5 min	Very rapid bedside screening; useful adjunctive tool	Limited sensitivity as a stand-alone test; influenced by motor or visual deficits
**HVLT-R (Hopkins Verbal Learning Test–Revised)**	Targeted neuropsychological test	Verbal learning, delayed recall, recognition memory	5–10 min	Highly sensitive to memory dysfunction; parallel versions available for repeated testing	Focused mainly on verbal memory; less informative for executive deficits
**COWAT (Controlled Oral Word Association Test)**	Targeted neuropsychological test	Phonemic verbal fluency, executive control, lexical retrieval	3–5 min	Sensitive to frontal lobe and dominant hemisphere dysfunction; brief and practical	Influenced by language proficiency, education, and cultural background
**TMT-A (Trail Making Test Part A)**	Targeted neuropsychological test	Psychomotor speed, visual scanning, attention	3–5 min	Sensitive to slowing of processing speed; quick to administer	Affected by visual impairment or motor dysfunction
**TMT-B (Trail Making Test Part B)**	Targeted neuropsychological test	Cognitive flexibility, set shifting, divided attention, executive functioning	5–10 min	One of the most sensitive brief measures of executive dysfunction	Strongly influenced by age, education, and motor speed
**Comprehensive Neuropsychological Battery**	Full assessment	Multidomain cognition, mood, behavior, functional impact	60–180 min	Gold standard for detailed cognitive profiling and rehabilitation planning	Time-consuming; may be impractical during intensive treatment or severe fatigue

**Abbreviations:** MoCA, Montreal Cognitive Assessment; MMSE, Mini–Mental State Examination; ACE-III, Addenbrooke’s Cognitive Examination III; CDT, Clock Drawing Test; HVLT-R, Hopkins Verbal Learning Test–Revised; COWAT, Controlled Oral Word Association Test; TMT, Trail Making Test.

## Data Availability

No new data were created or analyzed in this study. Data sharing is not applicable to this article.

## Abbreviations

ACE-III, Addenbrooke’s Cognitive Examination III; ASM, antiseizure medication; CDT, Clock Drawing Test; COWAT, Controlled Oral Word Association Test; GBM, glioblastoma; HGG, high-grade glioma; HRQoL, health-related quality of life; HVLT-R, Hopkins Verbal Learning Test–Revised; ICCTF, International Cognition and Cancer Task Force; IDH, isocitrate dehydrogenase; KPS, Karnofsky Performance Status; LGG, lower-grade glioma; MMSE, Mini–Mental State Examination; MoCA, Montreal Cognitive Assessment; MRI, magnetic resonance imaging; TMZ, temozolomide; TMT, Trail Making Test; TTFields, Tumor Treating Fields; WBRT, whole-brain radiotherapy.

## Appendix A

The literature search and study selection process are presented in Figure A1.
